# Mitochondrial Effects on Seeds of Cancer Survival in Leukemia

**DOI:** 10.3389/fonc.2021.745924

**Published:** 2021-10-07

**Authors:** Hend E. El-Shaqanqery, Rania Hassan Mohamed, Ahmed A. Sayed

**Affiliations:** ^1^ Genomics Program, Children’s Cancer Hospital Egypt, Cairo, Egypt; ^2^ Department of Biochemistry, Faculty of Science, Ain Shams University, Cairo, Egypt

**Keywords:** leukemia, leukemia stem cell, metabolism, mitochondria, mitophagy

## Abstract

The cancer metabolic alteration is considered a hallmark and fast becoming a road for therapeutic intervention. Mitochondria have been regarded as essential cell elements that fuel the metabolic needs of most cancer cell types. Leukemia stem cells (LSCs) are a heterogeneous, highly self-renewing, and pluripotent cell population within leukemic cells. The most important source of ATP and metabolites to fulfill the bioenergetics and biosynthetic needs of most cancer stem cells is the mitochondria. In addition, mitochondria have a core role in autophagy and cell death and are the main source of reactive oxygen species (ROS) generation. Overall, growing evidence now shows that mitochondrial activities and pathways have changed to adapt with different types of leukemia, thus mitochondrial metabolism could be targeted for blood malignancy therapy. This review focuses on the function of mitochondria in LSC of the different leukemia types.

## Introduction

Leukemia is described as an excessive division of blood-forming cells, resulting from failure of hematopoietic stem cell (HSC) death and abrogation of its differentiation (1, 2). Although these events occur in white blood cells, different blood cells are implicated in leukemia. Commonly, this kind of cancer is divided into two subtypes such as acute (speedy developing) or chronic (slow developing) leukemia (3). Hematologic disorders are still the most common cancer worldwide (4). Leukemia is one of the important causes of mortality in both developed and developing countries; as a result, it burdens high expenses to health scope (5). It has been predicted that deaths of about 50% younger patients and 90% older patients are because of acute myeloid leukemia (AML) or acute lymphoid leukemia (ALL), respectively (6, 7).

**Figure 1:**
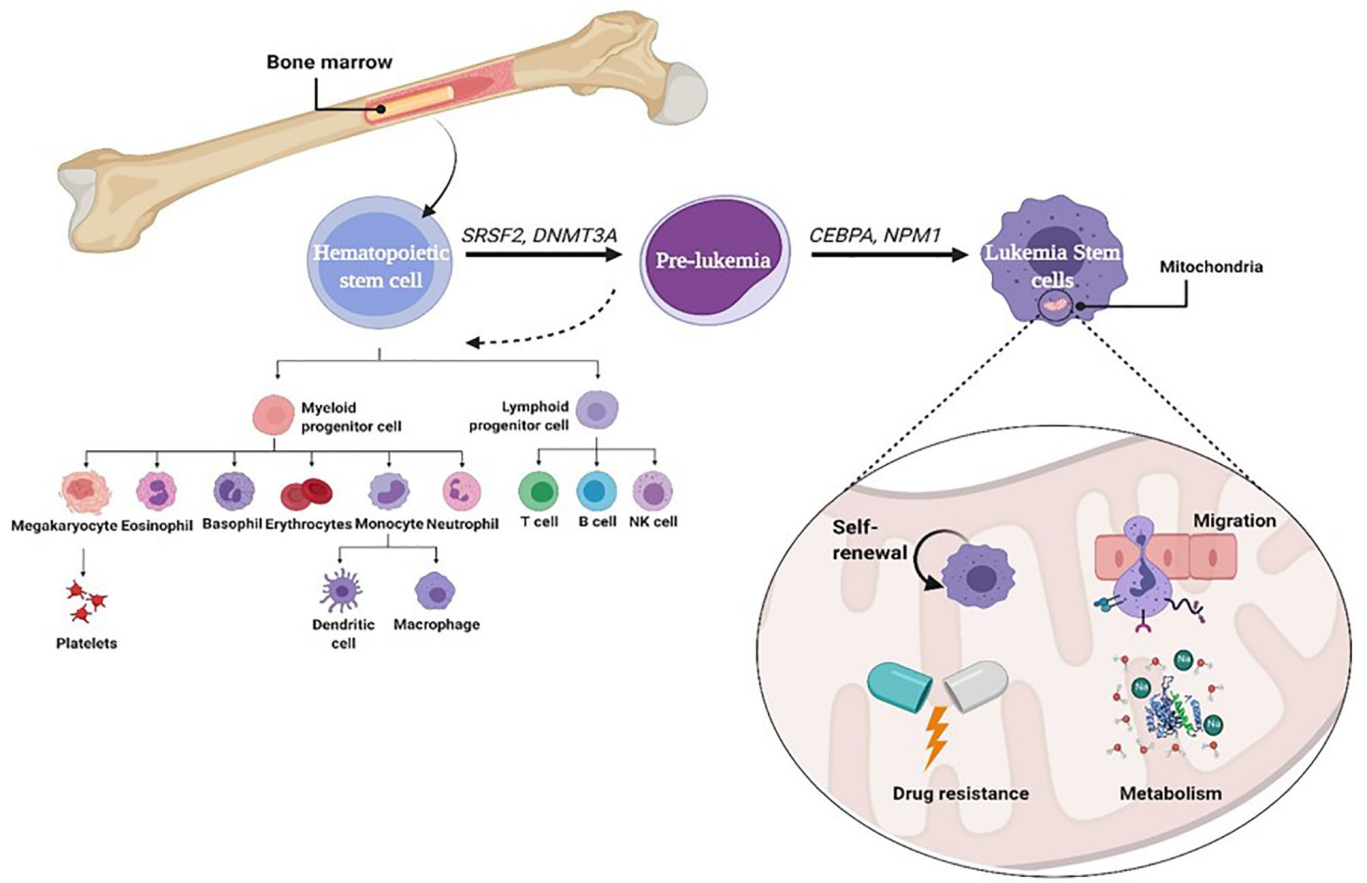
Role of mitophagy in leukemia stem cell.

Leukemic stem cells (LSCs) are a biologically and functionally defined entity. They are not always named because they arise from an ordinary stem cell but because they fulfill the standards used to define ordinary stem cells. LSCs are multipotent, incredibly proliferative, and self-renewing (8). Cancer stem cell (CSC) is called LSC when it exists in leukemia. It shares many characteristics with normal HSCs, including being CD38^+^ CD34^−^ stem cells (9). However, LSCs often upregulate the expression of other membrane markers such as CD123, TIM3, CD25, CD32, and CD96 that are absent from HSCs and vary among patients. Moreover, like HSCs and unlike leukemia myeloblasts, LSCs divide slowly (10). During normal progression from stem cell to progenitor cell to mature cell, mutations may probably arise at any stage, giving upward thrust to malignancy. Self-renewing HSCs that carry out genetic and epigenetic modifications can downregulate cell death and boost their self-renewal capability. A mutation in a normal stem cell can lead to the formation of a unit that could be considered an LSC. However, there may be experimental evidence suggesting that mutations in progenitor cells that do not have the complete characters of a stem cell can also lead to initiation and maintenance of the leukemic disease (11). A mutation of some genes (SRSF2, DNMT3A) in normal stem cells can lead to the formation of pre-leukemic stem cell (pre-LSC), while such pre-LSCs are capable of giving rise to healthy blood and immune cells. Additional mutations in CCAAT-enhancer-binding protein alpha (CEBPA) and nucleophosmin (NPM1) can cause a complete block in differentiation and thereby result in malignant expansion of aberrant progenitor cells that could be considered LSCs (**Figure 1**) (12). However, there may be experimental evidence suggesting that mutations in progenitor cells that do not have the complete characteristics of a stem cell can also lead to initiation and maintenance of the leukemic disease. The mutations of genes encoding mitochondrial enzymes, FMS-like tyrosine kinase-3 internal tandem duplication (FLT3-ITD) and isocitrate dehydrogenase (IDH), play a vital role in leukemia cell survival and chemoresistance. Recently, the IDH mutations offer the evidence for the relation between metabolism and leukemogenesis. These mutations play a pivotal role in the reprogramming of energetic metabolism in leukemic cells and in deregulation of ROS production. IDH mutations also enhance generation of 2-hydroxyglutarate (2-HG) instead of α-ketoglutarate (α-KG) (13). LSCs are often resistant to conventional chemotherapy, and their maintenance after therapy is a common reason for relapse. Therefore, it is vital to recognize the biological mechanisms that contribute to leukemia (14, 15). LSCs are characterized by a low rate of metabolism with a decreased basal reactive oxygen species (ROS) manufacturing as compared to bulk leukemic cells (16). These ROS-low LSCs are also unable to upregulate glycolysis after inhibiting oxidative phosphorylation (OXPHOS), which is in line with other reports showing that LSCs have unprecedented mitochondrial characteristics and an expanded sensitivity to strategies that block oxidative phosphorylation (17). Mitochondria play an essential role in metabolism, hypoxia, iron–sulfur clusters, cell differentiation, innate immunity, metabolism of amino acids, calcium, and heme biosynthesis (18). Furthermore, the redox balance of cells and the proapoptotic factor expression are managed by mitochondria to regulate cell death. Thus, there are critical functions of mitochondria inside the neoplastic phenotype, which include resistance to apoptosis, out of control proliferation, and metabolic reprogramming (15, 19).

CSCs ought to adapt their metabolism, especially by elevating nutrient uptake, to keep their uninhibited proliferation (20). Actually, CSC metabolism is not only an indirect by-product of proliferation but also an immediate reprogramming orchestrated through the use of oncogenic signals (21, 22). Studying the metabolic phenotype of LSCs would potentially clarify their mechanism of survival, persistence, and progression through the development of the disease. Understanding how they differ metabolically from HSCs can help better characterize any type of leukemia stem cell.

## Metabolic Flexibility of Leukemic Stem Cells

There are multiple levels of tumorigenesis process and mitochondrial biology interactions: tumorigenesis may start by direct signals from mitochondria (may be by mutations in mitochondrial DNA) or the alteration of mitochondrial functions of metabolism and bioenergetics by oncogenic signaling pathways. So, understanding the mechanisms of cancer-initiating cell metabolism will effectively aid to develop novel anticancer drugs targeting these aspects. **Table 1** summarizes the metabolic steps potentially implicated in the survival and therapy resistance of LSCs.

**Table 1:** Summary of the metabolic steps implicated in the survival and therapy resistance of LSCs.

Condition	Role	Ref.
**Glucose metabolism**	ATP production ROS production Cell stemness	(23)
**Glutamine Metabolism**	ATP production Regulate leukemia stem cell programing	(19, 24)
**Fatty acid metabolism**	ATP production Responsible for leukemia stem cell resistance to chemotherapy	(25)
**Hypoxia**	Maintaining leukemia stem cell stemness and drug resistance	(26, 27)
**Mitophagy**	Leukemia stem cell maintenance	(28)

LSC, leukemic stem cell; ROS, reactive oxygen species.

## Bioenergetics of Leukemic Stem Cells

LSCs are flexible and able to take advantage of more than one metabolic pathway to continue and survive. LSCs can use fatty acids and amino acids in addition to glucose to provide precursors to the tricarboxylic acid (TCA) cycle and to maintain mitochondrial metabolism (22). Most CSCs are dependent on and upregulate OXPHOS; hence, CSCs can be a target to mitochondrial inhibition. A study carried out in primary lymphocytes and CD34^+^ progenitors of patients with ALL suggests that, being an inhibitor of mitochondrial translation, tigecycline is capable of sensitizing them to multiplied apoptosis and would improve the levels of oxidative metabolism (29). Moreover, cytarabine-resistant AML cells are shown to be enriched in dormant LSCs and have higher functional mitochondrial mass, which is translated as increasing in OXPHOS levels with subsequent peak in ROS. Interestingly, although cytarabine was not effective, residual cells showed an increase in OXPHOS gene expression. In the study by Kuntz et al. (30), enriched and differentiated CD34^-^ cells were derived from patients with CML for metabolic analyses on each CD34^+^CD38^-^ stem cell, it has been proven that most primitive LSCs have higher mitochondrial efficacy than differentiated LSCs and normal CD34+CD38- cells. These studies show that primitive CML cells are reliant on oxidative metabolism for their survival (30). We will explain here the central carbon metabolism in LSC to fulfill their energy desires.

## Glucose Metabolism in Leukemic Stem Cells

The glycolytic pathway is the primary process in the metabolism of HSCs. Ordinarily, HSCs are energetically dormant with active glycolysis. In glycolysis, glucose is converted to pyruvate. In presence of oxygen, pyruvate can be metabolized to acetyl-CoA that is oxidized in the TCA cycle to drive OXPHOS and generation of ATP. LSCs often lack the ability to enhance glycolysis and therefore switch from anaerobic glycolysis to mitochondria-mediated OXPHOS as their major pathway to generate energy. LSCs rely upon OXPHOS for ATP generation instead of glycolysis and lactic acid fermentation, which gives a chance for ROS production, which can force cells out of quiescence and trigger programmed cell death pathways. Most ROS are generated in mitochondria *via* electron transport. As a result, LSCs respond to this action by upregulating autophagy, which is critical for the maintenance of stemness and the eradication of damaged mitochondria and production of ROS. This also upregulates the expression of the hypoxic response transcription factor [hypoxia-inducible factor 1-alpha (HIF-1-α], even in normoxia (23).

On the other hand, Song et al. (24) showed that bone marrow (BM) cells separated from AML patients without remission produce higher levels of HIF-1α and glucose transporter 1 (GLUT1), as well as hexokinase 2 (HK2) and lactate dehydrogenase (LDH), which are considered the main controlling stages of glycolytic flux, than those from patients with full or partial remission and healthy donors (31). By using metabolomics analysis in the study conducted by Bhanot et al. (31), UDP-P-glucose, a glycogen precursor to glucose, has been reported to be upregulated in AML independently of low glycogen levels. Additionally, adjustments in glucose metabolism have been linked to end-stage medical outcomes and drug resistance. Kreitz et al. (32) showed that there is excessive glycolytic level in blast AML reluctant to treatment. Also, it was suggested that myeloblast glycolytic rate may be an effective and effortless approach to decide the pretreatment prognosis of AML (25). Unfortunately, current studies are not sufficient to outline the LSC glycolytic phenotype, and extra studies are needed.

## Glutamine Metabolism in Leukemic Stem Cells

Metabolism of glutamine (glutaminolysis) is an alternative source of energy. Glutamine is the most considerable amino acid in circulation and can be supplied with the aid of adipose tissue as one of its fundamental sources to the LSCs (33). The mechanism by which tumor cells adjust the balance between glycolysis and oxidative metabolism to meet their energy needs is not fully understood.

It is known that the Warburg shift is an exquisite mark of the extra-proliferating cancer cells, which have an intact TCA cycle and gradually more dependent on glutamine metabolism compared to normal cells for ATP synthesis. Knoechel and Aster confirmed that a signal from the phosphoinositide 3-kinase (PI3K)–AKT pathway shifts NOTCH-dependent T-ALL cells from glutamine metabolism to aerobic glycolysis. Using murine models and the primary human T-ALL xenograft transplantation model, they showed that T-ALL cells with activating NOTCH1 mutations use glutamine as the main substrate for anaplerotic reactions that fuel the TCA cycle (34). In another study, MYC transcription factor and its “super enhancer” sequence have a critical function in hematopoietic malignancies by regulating the LSC programming (33). MYC promotes the uptake of essential amino acids (i.e., glutamine) through SLC7A5/SLC43A1 in lymphoma cells, which in turn stimulate the MYC translation and tumorigenesis (35, 36). Likewise, mutated IDH in AML-LSCs gains increased enzymatic feature to generate R-2-hydroxyglutarate (R2HG) from α-KG, rather than unmutated IDH, which catalyzes the conversion of isocitrate to α-KG. The subsequent accumulation of the oncometabolite R2HG inhibits the α-KG-dependent ten-eleven translocation (TET) protein family, which leads to DNA demethylation and consequently to tumorigenesis (36). The results of previous studies strongly recommend the glutamine metabolism key steps as a target for therapeutic strategies against LSCs.

## Fatty Acid Metabolism in Leukemic Stem Cells

The adipocytes have a central role in offering fatty acids to fulfill the high energy needs of the LSCs. Adipocytes can preserve energy as triglycerides, which at some point of lipolysis, it can be catabolized into glycerol and free fatty acids (FFAs). Therefore, adipocytes can also deliver FFA to cancer cells to meet their needs for lipid synthesis and energy.

Woolthuis et al. (37) showed that adipocytes offer FFAs as a source of energy to leukemia cells by a mouse model of blast-crisis CML. Surprisingly, they found a niche of LSCs in gonadal adipose tissue (GAT), and by using limiting-dilution transplantation techniques, it was found that GAT resident LSCs elevated in leukemia are similar to that derived from bone marrow. Likewise, the GAT-associated LSCs have elevated expression of the fatty acid transporter CD36. Gene expression analysis suggested that LSCs have a pro-inflammatory phenotype that will increase lipolysis to provide energy to LSCs with high levels of fatty acid oxidation (FAO) compared to highly differentiated progeny or normal HSCs. These characteristics control LSC quiescence and resistance to chemotherapy. A preceding study on primary human samples of AML recorded a subpopulation expressing CD36 in the CD34^+^ LSCs. This CD36^+^ phenotype was used as a poor prognosis indicator (38). In these cases, the CD36^+^ LSCs also showed an increase in FFAs and their subsequent oxidation, assuming that CD36 can adapt the LSC metabolism in at least one subgroup of human myeloid leukemia. Moreover, cytarabine-resistant AML cells located in quiescent LSCs have elevated levels of mitochondrial mass, which is translated into elevation of OXPHOS levels with high level in ROS. Surprisingly, although treatment with cytarabine was ineffective, residual cells exhibited high expression of OXPHOS genes collectively with increased FAO and upregulation of CD36 that can predict response to treatment in patients with AML (39). Tucci et al. (40) clarified that ALL cells accelerate adipocyte lipolysis and use the produced FFA to complement lipogenesis and *de novo* proliferation. In another study, Chronic Lymphocytic Leukemia (CLL) cells, in comparison with normal B lymphocytes, are able to catabolize lipids that allows usage of FFAs for oxidative respiration (41). FFAs also can interact with the nuclear receptor peroxisome proliferator-activated receptor α (PPARα). The interaction between FFAs and PPARα leads to generation of a complex that, much like a transcription factor, turns on the transcription of enzymes necessary for OXPHOS (42). Adipose tissue can also be protective for LSCs throughout stressful conditions, which includes drug treatment. Orgel et al. (43) showed that glutamine secreted by adipocytes protects leukemia cells from treatment with L-asparaginase. This is especially true, considering that L-asparaginase is used in the treatment of ALL, because leukemic lymphoblasts are quite sensitive to exogenous asparagine and glutamine depletion (41, 43).

This can show how LSCs can make the use of the surrounding microenvironment differential through several routes of metabolisms in different leukemia types. Furthermore, this suggests that targeting combined aspects of metabolism can be a complementary and powerful therapeutic strategy.

## Hypoxia and Hypoxia-Inducible Factors in Leukemic Stem Cells

Cells have a balanced antioxidant system to neutralize the extra ROS consisting of enzymatic antioxidants such as superoxide dismutase (SOD), glutathione peroxidases (GPxs), thioredoxin (Trx), and catalase (CAT) and non-enzymatic antioxidants to reduce the oxidative stress state (44). Human SOD can be classified into cytosolic CuZn-SOD, mitochondrial Mn-SOD, and extracellular SOD. The SOD seems to be the first line of defense against oxygen-derived free radicals as it can be rapidly induced in some conditions when exposed to oxidative stress as it catalyzes superoxide into oxygen and hydrogen peroxide (26). CAT can neutralize hydrogen peroxide through decomposing it into molecular oxygen and water. It is now well established that the mitochondria are the major producers of ROS and also the main targets of ROS. Immense accumulation of ROS and free radicals in mitochondria leads to elevated expression of Mn-SOD to inhibit oxidative damage in mitochondria. The accumulation of ROS will lead to mitochondrial permeability transition and disrupt the mitochondrial membrane stability (27). Disruption of mitochondrial outer membrane leads to cytochrome c release and other proapoptotic factors, such as serine protease OMI/HtrA2, Smac/Diablo, endonuclease G, and apoptosis-inducing factor (AIF), and consequently caspase activation and cell death (45). GPx family has antioxidative function at different cellular components: GPx1 is present ubiquitously in the cytosol and mitochondria, GPx2 is in the cytosol and nucleus, GPx3 is in the plasma, and GPx4 is membrane-associated and appears to protect membranes from oxidative challenge (46).

The Trx antioxidant system, composed of nicotinamide adenine dinucleotide phosphate (NADPH), thioredoxin reductase (TrxR), and Trx, is very important against oxidative stress as an endogenous antioxidant system. Trx antioxidants have a function in DNA and protein repair by reducing ribonucleotide reductase and methionine sulfoxide reductases. In addition, Trx systems have a significant role in the immune response (47). Homodimeric TrxR is a member of the pyridine nucleotide-disulfide oxidoreductase family, which includes TrxR, glutathione reductase (GR), TryR, alkyl hydroperoxide reductase, lipoamide dehydrogenase, and mercuric reductase. Trx and TrxR are the dimeric FAD-containing enzyme that catalyzes the NADPH-dependent reduction of the active-site disulfide in oxidized Trx (Trx-S2) to give a dithiol in reduced Trx [Trx-(SH)2] (48). Trx-(SH)2 is a hydrogen donor for ribonucleotide reductase and a disulfide reductase regulating thiol redox. Trx systems in cells can use the thiol and selenol groups to maintain redox level. Trx and its binding proteins [Apoptosis signal-regulating kinase 1 (ASK1) and TATA-box-binding protein 2 (TBP2)] appear to control apoptosis or metabolic states such as carbohydrate and lipid metabolism (49). Both GSH system and Trx system can defend against oxidative stress *via* the efficient removal of various ROS. Cytosolic Trx1 and mitochondrial Trx2 are the major disulfide reductases that affect cell proliferation and viability. The reduced/dithiol form of Trxs binds to ASK1 and inhibits its activity to induce apoptosis. When Trx is oxidized, it dissociates from ASK1 and apoptosis is induced (50). Non-enzymatic antioxidants like vitamin A or retinol, vitamin E, and vitamin C (51).

The role of hypoxia within the generation of LSCs remains debatable perhaps because of addressing hypoxia as a stemness factor in conflicting studies and the difference in duration and degree of hypoxia (52, 53). In any case, further investigations are required so as to clarify its impact on LSC maintenance and survival. Hypoxia by means of HIFs may drive powerful support and advancement through different pathways, for example, energy metabolism, cell cycle, and immune response. These physiological procedures can be upregulated or downregulated in malignancy. In AML, the presence of different oxygen levels in the BM permits upkeep of essential AML cells (28). The downregulation of HIF-2α or HIF-1α to a lesser degree was recorded by Rouault-Pierre et al. (54). Hypoxia promotes apoptosis and inhibits leukemic engraftment in human AML transplantation cells into mice. There are ongoing studies proving that keeping on the redox stability is fundamental for maintaining the stemness and drug resistance characteristics in most cancer cells (55, 56). The function of the PI3K/Akt/mammalian target of rapamycin (mTOR) signaling pathway in developing CSC traits of low apoptotic potential is reported to be partially through enhancement of the ROS elimination *via* CAT production downstream of nuclear localization of FoxOs and stimulation of the HIF-1α (57). In addition, Osellame et al. (58) validated that loss of mitochondrial outer membrane permeability is an indication for intrinsic apoptosis. These observations propose that HIF-2α or HIF-1α is important for development of LSCs and may possibly work as therapeutic targets for AML. Furthermore, the research by Velasco-Hernandez et al. (59) showed that the HIF-1α deletion does not influence the maintenance of AML in mice, which presents the inconsistencies in the role of HIF in AML. In any case, these variations may depend on the specific hereditary change that initiates the malignancy, again revealing the enormous heterogeneity of this disease. In addition, Vukovic et al. (60) developed a genetic model to investigate the effects of the deficiency in HIF-1α and HIF-2α during leukemogenesis. The model indicated that while HIF-2α had no effect on AML cell expansion in a murine model, it is significant in obstructing the development of LSC in malignancy. HIF-2α deletion enhances LSC differentiation, yet does not influence LSC maintenance of AML (60). In CML, Zhang et al. (61) recorded that deletion of HIF-1α prevents CML progression by inhibiting cell cycle and inducing LSC apoptosis. breakpoint cluster region-Abelson fusion gene (BCR-ABL) oncogene in CML-LSCs regulates HIF-1α to induce cell expansion. Regardless of whether HIF-1α has a function in the LSC survival in CLL is as yet obscure. In CLL, HIF-1α is regulated even under normoxia *via* downregulation of von Hippel–Lindau (VHL) protein, whose articulation is controlled by microRNAs (62). This system enables the formation of a complex (HIF-1α/p300/p-STAT3), which is responsible for the expression of the vascular endothelial growth factor (VEGF) (62). It was shown that upregulation of VEGF by HIF-1α assumes a significant role in the microenvironment controlling leukemic cell progression. In T-ALL, HIF-1α control promotes Wnt pathway through enhancing translation of β-catenin (63). Loss of HIF-1α diminishes the LSC recurrence without influencing the development and viability of leukemic cell mass.

## Mitophagy in Leukemic Stem Cells

Autophagy is defined as a self-digestion of the cell, wherein cytoplasmic materials, proteins (macroautophagy), damaged organelles like mitochondria (mitophagy), and lipids are segregated into vesicles, termed autophagosomes, for degradation and reusing. The quality and integrity of the mitochondria are basic to the typical elements of the mitochondria. Damaged mitochondria can be eliminated by mitophagy, which acts as a basic factor in the maintenance of stem cells. Various studies showed that stem cell self-renewal depends on mitophagy (64), illustrated in **Figure 1**. Decreasing Fis1 (mitochondrial division 1) in human LSCs weakens mitophagy, prompts cell cycle arrest, and disables self-renewal. It has been indicated that adenosine monophosphate-activated protein kinase (AMPK) enhances Fis1-dependent mitophagy, and AMPK inactivation mimics the mitophagy defect as a result of lack of Fis1 (14, 15). Mitochondrial dynamics likewise has a significant role in controlling mitophagy (65). LSCs indicated a constitutive activation of AMPK, a key player of controlling energy and mitochondrial homeostasis that arranges the initiation of autophagy and mitophagy through ULK1 activation (66). AMPK is a heterotrimeric serine/threonine kinase that phosphorylates plenty of cell substrates involved in various metabolic pathways through quick versatile reactions to various metabolites (66). AMPK mediates the crosstalk between various key cell signaling pathways regulating energy status, cell expansion, and autophagy through its negative control of the PI3K/AKT/mTOR pathway and its stimulatory impact on phosphorylation of ULK1 (67, 68). In addition, AMPK is a fundamental controller and sensor of cell energy status in mammalian cells. This kinase coordinates changes in the AMP/ATP and ADP/ATP ratios, adjusting the balance between ATP utilization and synthesis (69, 70)and acts to raise the catabolic processes and to diminish the anabolic processes to support intracellular energy homeostasis (71). It is stimulated by many conditions, such as nutrient deprivation (72), cell stresses (73), fasting or caloric limitation (74, 75), and nucleoside analogs like 5-aminoimidazole-4-carboxamide-1-β-D-ribofuranoside (AICAR) (76). In clinical trials, AMPK has been assessed for metabolic illness treatment and malignancies, including both hematopoietic cancers and solid tumors (77, 78), which showed that AMPK has a significant role in tumor regression. Notwithstanding, AMPK has a central role in controlling energy homeostasis and broadly associated with autophagy initiation (64, 79), life span, and tumor suppression (80, 81).

## Mitochondria and Leukemic Stem Cell Microenvironment

The connection between tumor cells and the tumor microenvironment (TME) impacts the phenotype of tumor cells (82). The TME involved different cell types including fibroblasts; immune, endothelial, and perivascular cells; and extracellular matrix (ECM) compartments, such as cytokines, growth factors, and extracellular vesicles. CSCs are thought to be inside or surrounded by the tumor environment maintaining the CSC “niche” and controlling its properties (83). CSC niche is thought to promote the formation of CSCs, keep the CSCs in stem-like state, shield them to resist the immune system, and induce the epithelial-to-mesenchymal transition (EMT), which improves tumor metastasis. Although the CSC niche has a major role in cancer growth, survival, and recurrence, it is still an obscure point needing more studies to solve its unique role in cancer dilemma.

## Bone Marrow Microenvironment Crosstalk With Leukemic Stem Cells

In light of the mentioned behavior of the LSC, the BM metabolic microenvironment supports the development of the leukemia cell stemness and pre-metastatic niche. Interestingly, Marlein et al. (15) showed that NADPH oxidase 2 (NOX2) generates superoxide, which causes BM stromal cells to move mitochondria through AML-derived tunnel nanotubes to AML blasts. Indeed, the quiescent CLL cells cultured in the presence of three distinctive stromal cell lines exhibit higher OXPHOS when compared to CLL cells cultured alone. A group of 28 CLL patient-derived cells cocultured with BM could stimulate natural killer (NK) cells, M2-10B4 fibroblasts, or HS-5 stromal cells to feature the significance of considering cell–cell communications (84). Cai et al. (85) revealed that culturing the T-ALL cells with mesenchymal stem cells (MSCs) decreases their mitochondrial ROS levels and initiate a Warburg-like shift that is portrayed by an expansion in glucose uptake and generation of lactate with decrease in ATP synthesis and mitochondrial membrane potential. Moreover, T-ALL cells cocultured with MSCs have adjusted mitochondrial morphology because of the extracellular signals involved in the phosphorylation of the factor, the protein related to dynamin 1 (Drp1) at residue S616. Consistently, phosphorylated Drp1 expression in S616 retained mitochondrial ROS levels, mitochondrial dynamics, metabolic exchange, and chemoresistance in T-ALL cells cocultured with MSCs (85). In addition, the BM mesenchymal stromal cells increase the metabolism and proliferation of their CML cell neighbors by secreting placental growth factor (86, 87). Moreover, the BM stroma uses multiple metabolic regulatory strategies to enhance the stemness traits of the leukemic cells, such as the induction of resistance of ALL cells to asparaginase treatment by secreting high concentrations of asparagine from MSCs (88). Conversion of cystine to cysteine by the BM stroma also protects CLL cells from the oxidative damage (89). The activated p53 pathway and secretion of inflammatory mediators from BM stromal cells activate the initiation of leukemia *via* activation of TLR4, which induces the mitochondria hyperpolarization, ROS production, and DNA double-strand breaks in hematopoietic stem and progenitor cells (HSPCs) (90). On the other hand, the BM stromal cells protect the LSCs from chemotherapy by upregulation of mitochondrial proteins [i.e., B-cell lymphoma 2 (BCL2) and Uncoupling Protein 2 (UCP2)] to uncouple the leukemic mitochondria and support the glycolytic pathways (91, 92). Another regulator for the metabolic niche of the LSC is the microRNA; LIN28B has been proven to enhance the stemness of the LSC by repression of Let-7, which regulates the insulin-like growth factor 2 mRNA-binding protein 1 (IGF2BP1) (93). Leukemia cells need metabolic adaptation not only for its survival and growth but also to educate the BM milieu to support the LSC reprogramming. Leukemic cells induce a state of insulin resistance in the surrounding cells by increasing the IGFBP1 production from the adipose tissue to save sufficient glucose level for LSC usage (94). After depletion of glucose in the BM microenvironment, the AML cells are encouraged to consume fructose as a source of energy by upregulation of GLUT5 on leukemic cells (95). Moreover, leukemic cells in contact with adipocytes enhance lipolysis by increasing the Fatty Acid-Binding Protein 4 (FABP4) to provide the fatty acids, which are essential for LSC survival (96).

## Immunometabolic Regulation in Leukemic Stem Cells

CSCs exhibit metabolic flexibility not only to promote the biosynthetic and bioenergetic needs of tumor malignancy but also to evade the antitumor immunity. The increased consumption of nutrients for the high metabolic competitive CSC deteriorates the metabolic resources of the immune cells in the TME (97, 98). The tumor infiltrated immune cells can act as protumor or antitumor, but both mechanisms are influenced by the metabolic activities of leukemic cells of AML (99). In support of that, the high consumption of glucose and amino acids in cancer cells downregulates the cytotoxic T and NK cell energy metabolism and subsequently their activation and effector functions (100). Also, lactate production suppresses monocyte activation (101) while increasing the tumor-promoting cytokine expression [i.e., interleukin (IL) -23] (102). Also, oxidative stress metabolic products can alter the functions of regulatory T cells (T regs) and myeloid dendritic cells (103, 104). In line with the mentioned immune-metabolic adaptation and to feed the gluttonous needs of the CSCs, leukemia cells secrete several inflammatory mediators such as IL-6, IL-1β, tumor necrosis factor (TNF)α, and granulocyte colony-stimulating factor (G-CSF), along with the endothelial granulocyte-macrophage colony-stimulating factor (GM-CSF), to enhance vasculogenesis for supplying the AML LSC with the essential metabolites for its growth and proliferation (85). Also, the AML blasts suppress T-cell proliferation and enhance the polarization into the M2 suppressive monocyte by secreting high levels of arginase II (88).

Although ROS is a direct effector for killing pathogens *via* innate immune cells, ROS has a crucial role as an immunosuppressive agent helping CSCs/LSCs to evade the immune system and to enhance cancer stemness and anti-leukemic lymphocyte resistance (105, 106). One suggested mechanism for its role in immune evasion, ROS released from these malignant cells is able to induce apoptosis and to reduce the cytokine production of the anti-leukemic lymphocytes. The CSCs maintain their redox balance to keep their stemness traits, survival, and immune evasion. Hence, AML cells with low ROS level represent more CSC and quiescent characteristics (56, 57). Therefore, antioxidants are considered double-edged weapons. They can act as tumor suppressors by decreasing the ROS apoptotic effect on NK and T cells against leukemic cells. On the other hand, antioxidants act as cancer stemness inducers through targeting of ROS-mediated signaling. For example, the GSH precursor N-acetylcysteine can reduce the effect of ROS in Acute myeloid leukemia stem cells (AMLSCs), which resists the niclosamide antineoplastic and apoptotic effect through inhibition of TNFα-induced nuclear factor (NF)-κB activation and increase of the intracellular ROS levels (107).

Signal transducer and activator of transcription (STAT3) can modulate the immune TME to maintain CSC characteristics and renewal. This can be by promoting the functions of MDSC *via* increased ROS and NOX2 expression. Some anticancer candidates such as fluorinated β-amino-ketone and AZD9159, antisense oligonucleotides, are used to inhibit the T regs, MDSC, and tumor growth *via* suppression of STAT3 expression and cascade. One other way is by increasing the expression of HIF-1α, as STAT3 is an upstream transcription factor for HIF-1α, in cancer cells and myeloid cells in the TME, which is critical for immunosuppression and tumor immune evasion (107). HIF-1α, STAT3, and CBP/p300 are known as transcriptional complex components that regulate the response to hypoxia in cancer. HIF-1α/p300/p-STAT3 axis enhances the immune evasion mechanisms of CSC by inhibition of T-cell proliferation, activation, and induction of T regs *via* VEGF upregulation (108). In line with this, HIF-1α/p300/p-STAT3 axis is considered a therapeutic target to eradicate cancer progression. Triptolidenol-1 (LB-1) was used to inhibit the HIF-1α activity, increase its degradation, and suppress the connection between HIF-1α, p-STAT3, and p300. Also, TEL03 is used as a phosphorylation inhibitor for STAT3, which suppresses HIF-1α expression (109). On the other hand, HIF-1α regulates the expression of natural killer group 2 member D (NKG2D) ligands to enhance tumor immunosurveillance by NK and γδ T cells. HIF-1α downregulation increases the shedding of soluble NKG2D ligands (sNKG2D) such as soluble Major Histocompatibility class I polypeptide–related sequence A (sMICA) to enhance the tumor immune evasion (110). Furthermore, it was reported that STAT3 can work as a tumor suppressor by inhibiting aerobic glycolysis of tumor cells, which decreases glucose consumption, lactate production, and expression of HIF-1α target genes in the tumor cells. In such a way, STAT3 and HIF-1α can mediate tumor immunity and immune evasion (111). So, finding the balance between the pros and cons of targeting HIF-1α and STAT3 can be a direction to solve the problems associated with tumor therapy through modulating the immune response.

The hematopoietic stem cell transplantation (HSCT) therapy of leukemia has been greatly improved due to the accurate typing and selection of the donors. However, there is a high level of relapse and low rates of survival. This challenge is supported by BM microenvironment, which can be reprogrammed by LSC to enhance the stemness characteristics of both HSCs and LSCs and promote leukemia initiation (112). Although the metabolic immunoregulation of LSCs is not completely and directly addressed so far, Du et al. (113) have investigated the metabolic axis of the hematopoietic progenitor microenvironment to suppress the anti-leukemic immunity. They connected inflammation, metabolism, and cancer immunity through cyclooxygenase (COX)2/prostaglandin (PG)/The nuclear orphan receptor 4A (NR4A)/Wingless/int1 (WNT) immunometabolism-regulatory axis. The role of pro-inflammatory (COX2) upregulation and its products, PGs, has been reported in hematological malignancies (114). The elevated COX2 and PGs in AML-MSCs in the BM niche increased the expression of NR4A transcription factors and the WNT signaling pathways, which has been known to be associated with many CSC traits (115). Inhibition of this axis could ameliorate anti-leukemic reactive T effector cells (113). All the previous links suggest the possibility of immunotherapeutic targeting through metabolic routes.

## Conclusion and Future Prospective

LSC is a mutated stem cell with normal stemness characteristics and also can differentiate to give rise to a cancerous hematopoietic lineage that accumulates the immature blast cells. Mitochondria not only is a main player in the LSC survival and malignancy development but also change the TME to keep the LSC alive. It regulates the redox status, bioenergetics, nutritional dependence, and metabolic products according to the available substrates, as well as modifies the surrounding immune milieu of the tumor. In this review, we highlighted the role of mitochondria in the adaptation of the demanding LSCs to the microenvironment and the development of their stemness traits. LSCs are resilient to use a range of sources such as glucose, amino acids, and fatty acids as precursors for TCA cycle. These leukemia seeds might easily switch between OXPHOS and glycolysis to provide their needs of energy, biogenesis, and drug resistance. Regulation of essential amino acid transporter expression and glutamine metabolism is not only a source of ATP but also considered an immune evasion strategy of LSCs. The crosstalk between adipocytes and LSCs by delivering fatty acids resulting from lipolysis is an unescapable factor for LSC survival. Also, keeping the ROS and oxygen gradient allows the maintenance of the LSCs. Here, we shed light on LSC flexibility to gain their needs of energy, which offers a new therapeutic strategy to target the metabolic reprogramming of LSCs, maybe specifically, in the different types of leukemia. We also focused on mitophagy and AMPK as an initiator of autophagy and mitophagy. AMPK is considered a potential therapeutic target to control the progression of LSCs in different types of leukemia. Moreover, the disruption of the interaction between the BM stroma and LSC may be of great importance to eliminate LSCs and improve HSCT outcomes in different types of leukemia.

## Author Contributions

HE-S and RM performed the literature search and drafted the manuscript. HE-S drew the figure. AS edited and revised the manuscript. All authors contributed to the article and approved the submitted version.

## Funding

This study was supported by the Department of Basic Research, Children’s Cancer Hospital Egypt 57357 (CCHE 57357).

## Conflict of Interest

The authors declare that the research was conducted in the absence of any commercial or financial relationships that could be construed as a potential conflict of interest.

## Publisher’s Note

All claims expressed in this article are solely those of the authors and do not necessarily represent those of their affiliated organizations, or those of the publisher, the editors and the reviewers. Any product that may be evaluated in this article, or claim that may be made by its manufacturer, is not guaranteed or endorsed by the publisher.
